# Supplementary material for: Evaluating Food Packaging Waste in Schools: A Systematic Literature Review
Source: Int J Environ Res Public Health. 2022 May 5;19(9):5607. doi: 10.3390/ijerph19095607 (PMC9101714; doi:10.3390/ijerph19095607)
Supplement: Supplementary file 1 [file ijerph-19-05607-s001.zip › Figure-S1_WASTE-Instrument_Final.pdf]

Figure S1: Waste Audit for Sustainable Transitions and Evaluations (WASTE) Instrument

School Name$_{Q1}$:___________________________ **School ID#$_{Q2}$:** ________________________ **Data Collector$_{Q3}$:** _________________

Date$_{Q4}$: __ __ /__ __ /__ __ **Lunch Start Time$_{Q5}$:** __ __: __ __ am/pm **Grade of Students$_{Q6}$:**___

Directions: For a pre-sort evaluation, collect the weight and/or volume of every waste stream (‘waste bin receptacle analyzed’). For a post-sort evaluation, record the ‘waste bin receptacle analyzed’ and sort each disposable foodware and packaging element (solid waste item) into a separate bucket that is lined with a 6-gallon bag (e.g. all utensils in one bucket, all napkins in a different one). If a bucket becomes full, tie off the bag and insert another bag into the bucket, then keep sorting. Record the ‘individual item weight’ of each solid waste items from 3 separate samples of that item. Record the aggregate weight (weight of bags) of all items. Make sure to tare the bucket and bag before weighing. Record the number of bags (round to the nearest 0.25) per solid waste item. Tally the number of each item collected. Collect one of each solid waste items (including food serviceware) and place in a bag labeled with the school name, school ID, data collector, and date. Determine the volume collection timing (before waste sort, after it, or N/A) and its process (neatly stacked, tossed into a bin, or N/A).

Photograph$_{Q7}$: ☐$_1$ A typical meal (beverage/food containers or wrapping, napkins, utensils) ☐$_2$ Waste bin receptacles ☐$_3$ Waste bin signage

Waste Bin Receptacles Types$_{Q8}$: (L) Landfill#$_1$_____ (R) Recycling #$_2$_____ (C) Compost#$_3$_____ (O) Other#:$_4$____ Other Describe:$_5$__________

Where Material Collected$_{Q9}$: Kitchen/ back of house$_1$_____ Cafeteria/ front of house$_2$_____

Meal Type$_{Q10}$: Breakfast$_1$_____ Lunch$_2$_____ Snack$_3$_____ Dinner$_4$_____

Volume Data Collection Timing$_{Q11}$: Before waste sort$_1$_____ After waste sort$_2$_____ N/A$_3$_____

Volume Data Collection Process* $_{Q12}$: Neatly stacked$_1$_____ Tossed into a bin$_2$_____ N/A$_3$_____

Scale Calibration (500g)$_{Q13}$: __ __ __.__ (g)

**Solid Waste Item Guide:**	**Material Type Guide:**
(BC) Beverage Container: carton, pouch, cup<br>(FC) Food Container: tray, boat, plate, bowl<br>(W) Wrapping: film plastic lid, tin foil wrap<br>(U) Utensil: fork, spork, spoon, knife, chopsticks<br>(N) Napkin<br>(S) Straws<br>(O) Other	(CP) Compostable Plastic: must say “compostable” or inside the chasing arrows symbol is #7/PLA, #7/Other, or #0<br>(CF) Compostable Paper: napkin, paper towel, or fiber item that is labeled “compostable”<br>(P) Plastic: inside the chasing arrows symbol is #1-6 or plastic without a chasing arrows symbol<br>(LP) Lined Paper: milk containers, paper cups, food containers<br>(O) Other: foil, paper-lined foil, waxed paper, tamale husks, etc.

Notes (e.g. two different food items have the same packaging, deviations from protocol, waste bin signage or location abnormalities, etc.):$_{Q14}$

*In collecting volume data, it is possible to stack the items neatly in a bag then calculate the volume or toss them into a bag to emulate typical discard behavior then calculate the volume

Waste Bin Receptacle Analyzed	Solid Waste Item	Description	How Provided	Material Type		Individual Item Weight			Aggregate Weight	Aggregate Volume	Spec. Collected
**(L) landfill (R) recycling (C) compost (O) other (N/A)** if waste not analyzed from a bin. Q15	**(BC) (FC) (W) (U) (N) (S) (O)** Q16	**Include what solid waste item was used for.** If there are items that have identical packaging combine them and and note that here (e.g. chicken pot stickers and bean/cheese pupusa) Q17	**(A) automatically (R) by request (SS) self-serve** Q18	**(CP) (CF) (P) (LP) (O)** Q19.1	**Resin Code / # in recycling symbol** (put NA if not on packaging) Q19.2	**Sample 1 (g)** Q20.1	**Sample 2 (g)** Q20.2	**Sample 3 (g)** Q20.3	**Total weight of all collected items** (g) Q21	**Total number of __-gallon bags** (round to nearest 0.25) Q22	**Check box** Q23
L	*U*	*Fork used for multiple food items*	*A*	*P*	*1*	*2.5*	*2.4*	*2.5*	__ __ __.__ g<br>__ __ __.__ g<br>__ __ __.__ g<br>__ __ __.__ g	*10.25*	√
									__ __ __.__ g<br>__ __ __.__ g<br>__ __ __.__ g<br>__ __ __.__ g		
									__ __ __.__ g<br>__ __ __.__ g<br>__ __ __.__ g<br>__ __ __.__ g		
									__ __ __.__ g<br>__ __ __.__ g<br>__ __ __.__ g<br>__ __ __.__ g		
									__ __ __.__ g<br>__ __ __.__ g<br>__ __ __.__ g<br>__ __ __.__ g		
									__ __ __.__ g<br>__ __ __.__ g<br>__ __ __.__ g<br>__ __ __.__ g		
									__ __ __.__ g<br>__ __ __.__ g<br>__ __ __.__ g<br>__ __ __.__ g		

Solid Waste Guide: (BC) Beverage Container: carton, pouch, cup (FC) Food Container: tray, boat, plate (W) Wrapping (U) Utensil (N) Napkin (S) Straws (O) Other
Material Type Guide: (CP) Compostable Plastic: must say "compostable" or inside the chasing arrows symbol is #7/PLA, #7/Other, or #0 (CF) Compostable Fiber: napkin, paper towel, or fiber item that is labeled "compostable" (P) inside the chasing arrows symbol is #1-6 or plastic without a chasing arrows symbol (LP) Lined Paper: milk containers, paper cups, food containers (O) Other: foil, paper-lined foil, waxed paper, tamale husks, etc.

School ID#$_{Q2}$: __________________ **Data Collector$_{Q3}$:** __________________ **Date$_{Q4}$:** __ __ /__ __ /__ __

Waste Bin Receptical Analyzed	Solid Waste Item	Description	How Provided	Material Type		Individual Item Weight			Aggregate Weight	Aggregate Volume	Spec. Collected
**(L) landfill<br>(R) recycling<br>(C) compost (O) other<br>(N/A)** if waste not analyzed from a bin.<br>Q15	**(BC) (FC)<br>(W) (U)<br>(N) (S) (O)**<br>Q16	**Include what solid waste item was used for.**<br>If there are items that have identical packaging combine them and and note that here (e.g. chicken pot stickers and bean/cheese pupusa)<br>Q17	**(A) automatically<br>(R) by request<br>(SS) self-serve**<br>Q18	**(CP)<br>(CF)<br>(P)<br>(LP)<br>(O)**<br>Q19.1	**Resin Code / # in recycling symbol**<br>(put NA if not on packaging)<br>Q19.2	**Sample 1 (g)**<br>Q20.1	**Sample 2 (g)**<br>Q20.2	**Sample 3 (g)**<br>Q20.3	**Total weight of all collected items**<br>(g)<br>Q21	**Total number of __-gallon bags** (round to nearest 0.25)<br>Q22	**Check box**<br>Q23
									__ __ __.__ g<br>__ __ __.__ g<br>__ __ __.__ g<br>__ __ __.__ g		
									__ __ __.__ g<br>__ __ __.__ g<br>__ __ __.__ g<br>__ __ __.__ g		
									__ __ __.__ g<br>__ __ __.__ g<br>__ __ __.__ g<br>__ __ __.__ g		
									__ __ __.__ g<br>__ __ __.__ g<br>__ __ __.__ g<br>__ __ __.__ g		
									__ __ __.__ g<br>__ __ __.__ g<br>__ __ __.__ g<br>__ __ __.__ g		
									__ __ __.__ g<br>__ __ __.__ g<br>__ __ __.__ g<br>__ __ __.__ g		
									__ __ __.__ g<br>__ __ __.__ g<br>__ __ __.__ g<br>__ __ __.__ g		

Solid Waste Guide: (BC) Beverage Container: carton, pouch, cup (FC) Food Container: tray, boat, plate (W) Wrapping (U) Utensil (N) Napkin (S) Straws (O) Other
Material Type Guide: (CP) Compostable Plastic: must say "compostable" or inside the chasing arrows symbol is #7/PLA, #7/Other, or #0 (CF) Compostable Fiber: napkin, paper towel, or fiber item that is labeled "compostable" (P) inside the chasing arrows symbol is #1-6 or plastic without a chasing arrows symbol (LP) Lined Paper: milk containers, paper cups, food containers (O) Other: foil, paper-lined foil, waxed paper, tamale husks, etc.

School ID#$_{Q2}$: __________________ **Data Collector$_{Q3}$: __________________** **Date$_{Q4}$: __ __ /__ __ /__ __**

Waste Bin Receptical Analyzed	Solid Waste Item	Description	How Provided	Material Type		Individual Item Weight			Aggregate Weight	Aggregate Volume	Spec. Collected
**(L) landfill (R) recycling (C) compost (O) other (N/A)** if waste not analyzed from a bin. Q15	**(BC) (FC) (W) (U) (N) (S) (O)** Q16	**Include what solid waste item was used for.** If there are items that have identical packaging combine them and and note that here (e.g. chicken pot stickers and bean/cheese pupusa) Q17	**(A) automatically (R) by request (SS) self-serve** Q18	**(CP) (CF) (P) (LP) (O)** Q19.1	**Resin Code / # in recycling symbol** (put NA if not on packaging) Q19.2	**Sample 1 (g)** Q20.1	**Sample 2 (g)** Q20.2	**Sample 3 (g)** Q20.3	**Total weight of all collected items** (g) Q21	**Total number of __-gallon bags** (round to nearest 0.25) Q22	**Check box** Q23
									__ __ __.__ g<br>__ __ __.__ g<br>__ __ __.__ g<br>__ __ __.__ g		
									__ __ __.__ g<br>__ __ __.__ g<br>__ __ __.__ g<br>__ __ __.__ g		
									__ __ __.__ g<br>__ __ __.__ g<br>__ __ __.__ g<br>__ __ __.__ g		
									__ __ __.__ g<br>__ __ __.__ g<br>__ __ __.__ g<br>__ __ __.__ g		
									__ __ __.__ g<br>__ __ __.__ g<br>__ __ __.__ g<br>__ __ __.__ g		
									__ __ __.__ g<br>__ __ __.__ g<br>__ __ __.__ g<br>__ __ __.__ g		
									__ __ __.__ g<br>__ __ __.__ g<br>__ __ __.__ g<br>__ __ __.__ g		

Solid Waste Guide: (BC) Beverage Container: carton, pouch, cup (FC) Food Container: tray, boat, plate (W) Wrapping (U) Utensil (N) Napkin (S) Straws (O) Other
Material Type Guide: (CP) Compostable Plastic: must say "compostable" or inside the chasing arrows symbol is #7/PLA, #7/Other, or #0 (CF) Compostable Fiber: napkin, paper towel, or fiber item that is labeled "compostable" (P) inside the chasing arrows symbol is #1-6 or plastic without a chasing arrows symbol (LP) Lined Paper: milk containers, paper cups, food containers (O) Other: foil, paper-lined foil, waxed paper, tamale husks, etc.

School ID#$_{Q2}$: __________________ **Data Collector$_{Q3}$: __________________** **Date$_{Q4}$: __ __ /__ __ /__ __**
