# Supplementary material for: Evaluating Food Packaging Waste in Schools: A Systematic Literature Review
Source: Int J Environ Res Public Health. 2022 May 5;19(9):5607. doi: 10.3390/ijerph19095607 (PMC9101714; doi:10.3390/ijerph19095607)
Supplement: Supplementary file 1 [file ijerph-19-05607-s001.zip › Table-S1_Studies_Final.pdf]

**Table S1**: **All recorded characteristics of reviewed studies**. All recorded characteristics of studies (n=24) included in the systematic review of methods used to evaluate school foodservice packaging waste, sorted by method.

| Author(s) | Year | Method(s) | Objective and Subjective Measures | Waste Bin Types | Observational / Experimental (Study Design) | Country (State/ Province) | School Type (Number of Schools Studied) | Environ-ment Assessed | Study Participants | Number of Study Partici-pants | Racial and Socio-economic Demo-graphics | Interven-tion | Interven-tion Period | Waste Audit Data Collection Period | Publica-tion Type |
|---|---|---|---|---|---|---|---|---|---|---|---|---|---|---|---|
| **Arazo RA [38]** | 2015 | waste audit (sorted + weighed) | food packaging waste categorized: paper and paper products, hard plastics, soft plastics, glasses, metals, woods, food leftovers, yard, textile, inorganic, hazardous, special wastes | trash | observational (cross-sectional) | Philippines (--) | college/ university (1) | whole school | students, staff, and administra-tors | -- | -- | -- | -- | 4-week sampling period | peer-reviewed |
| **Castrejon A [34]** | 2008 | waste audit (sorted + weighed) | food packaging waste categorized: mixed paper, food scrap and yard waste, cans/ bottles, trash | trash, recycling (mixed paper), recycling (cans/bottles), compost (food scraps and yard waste) | observational (cross-sectional) | US (CA) | elementary school (3) | whole school | students | 940 | geographic information; ethnicity | -- | -- | one 10-week waste audit | master's thesis |

| | | | | | | | | | | | | | | | |
|---|---|---|---|---|---|---|---|---|---|---|---|---|---|---|---|
| **Felder MAJ [41]** | 2001 | waste audit (sorted + weighed) | food packaging waste categorized: animal bedding, animal waste, cloth, food, wood, glass, paper, plastic (#1, 2, 5), plastic (#3, 4, 6, 7) metal (aluminum), metal (ferrous), miscellaneous | trash, recycling, compost (food scraps) | observational (cross-sectional) | Canada (British Columbia) | college/ university (1) | whole school | students, staff, and administrators | -- | -- | -- | -- | three-plus 1-day waste audits per activity area | peer-reviewed |
| **Gallardo A [39]** | 2016 | waste audit (sorted + weighed) | food packaging waste categorized: plastics (PET, HDPE, LDPE, PP and PS), ferrous metals, nonferrous metals, clean and dirty paper, clean and dirty cardboard, Tetra Brick cartons, glass, organic matter, sanitary cellulose, rubber and | trash | observational (cross-sectional) | Spain (--) | college/ university (1) | whole school | students, staff, and administrators | 17,792 | -- | -- | -- | two 1-day waste audits | peer-reviewed |

| | | | | | | | | | | | | | | | |
|---|---|---|---|---|---|---|---|---|---|---|---|---|---|---|---|
| | | | leather, toxic and hazardous waste, inert waste | | | | | | | | | | | | |
| **Hahn NI [14]** | 1997 | waste audit (sorted + weighed) | food packaging waste categorized: carton, pouch, cardboard, metal cans, compost | trash | experimental (pretest-posttest) | US (LA) | K-12 (6) | cafeteria | students | -- | -- | waste-reduction or recycling adoption program | -- | -- | peer-reviewed |
| **James L [43]** | 2015 | waste audit (sorted + weighed) | food packaging waste categorized: recycling, trash, compost | trash, recycling, compost (food scraps) | experimental (cross-sectional) | US (WA) | elementary school (n/a) | whole school | students, staff, and administrators | 847 | ethnicity | waste-reduction or recycling adoption program | 1 month - 1 year | three 1-day waste audits | peer-reviewed |
| **Ramamoorthy R [37]** | 2019 | waste audit (sorted + weighed) | food packaging waste categorized: food, paper, silt/ soil/ mud, plastic, wood/ glass/ metal/ textile, clinical/ sanitary, e-waste, other | student waste, campus waste | observational (cross-sectional) | India (--) | K-12 (487) | whole school | students | 255,248 | geographic information | -- | -- | one 5-day waste audit | peer-reviewed |

| | | | | | | | | | | | | | | | |
|---|---|---|---|---|---|---|---|---|---|---|---|---|---|---|---|
| **Ravenelle J [51]** | 2018 | waste audit (sorted + weighed) | food packaging waste categorized: trash, recycling, food, liquid | trash, recycling, compost (food scraps and liquid waste) | experimental (cross-sectional) | US (ME) | elementary school (3) | cafeteria | students | 1,669 | geographic information; FRPM | waste-reduction or recycling adoption program | <1 month | three 1-day waste audits | peer-reviewed |
| **Schumpert K [48]** | 2012 | waste audit (sorted + weighed) | food packaging waste categorized: comingled containers, paper, cardboard, food waste, non-recyclable paper | trash, recycling, compost (food scraps) | experimental (cross-sectional) | US (CO) | K-12 (31) | whole school | students, staff, and administrators | -- | -- | waste-reduction or recycling adoption program; formal education | 1 month - 1 year | -- | report |
| **Schupp CL [52]** | 2018 | waste audit (sorted + weighed) | food packaging waste categorized: reusable food items, compost, recycling, trash | trash, recycling, compost (food scraps) | experimental (cross-sectional) | US (WA) | K-12 (18) | cafeteria | students, staff, and administrators | 18 | FRPM | formal education | >1 year | one 18-day waste audit | peer-reviewed |
| **Hollingsworth M [50]** | 1995 | waste audit (sorted + weighed + volume) | food packaging waste categorized: food, oil/suet, cardboard, paper, metal, plastic, glass, milk component, plate waste, | trash, recycling | experimental (cross-sectional) | US (LA) | K-8 (7) | cafeteria | staff and administrators | 6,513 | -- | waste-reduction or recycling adoption program | <1 month | two 10-day waste audits | peer-reviewed |

| | | | | | | | | | | | | | | | |
|---|---|---|---|---|---|---|---|---|---|---|---|---|---|---|---|
| | | | miscellaneous | | | | | | | | | | | | |
| **Baca J [35]** | 2011 | question-naire | food packaging waste categorized: recycling, trash, compost | trash, recycling, compost | observational (cross-sectional) | US (Multiple States) | K-12 (n/a) | cafeteria | staff and administra-tors | 79 | -- | -- | -- | -- | master's thesis |
| **Chan TC [46]** | 2013 | question-naire | level of MSW practices | n/a | experimental (cross-sectional) | US (GA) | K-12 (30) | whole school | students, staff, and administra-tors | 30 | ethnicity; FRPM | formal education | -- | -- | report |
| **Fleckenstein RM [47]** | 2016 | question-naire | food packaging waste categorized: paper, plastic, styrofoam, metal | trash, recycling, compost | observational (cross-sectional) | US (NJ) | K-12 (34) | whole school | staff and administra-tors | 75 | geographic information | -- | -- | 1 day | doctoral dissertation |
| **Iojă CI [36]** | 2012 | question-naire | level of food packaging knowledge | -- | observational (cross-sectional) | Romania (--) | K-12 (457) | whole school | students, staff, and administra-tors | 218 | -- | -- | -- | -- | peer-reviewed |
| **Vitamog AT [42]** | 2012 | question-naire | level of MSW practices | -- | observational (cross-sectional) | Philippines (--) | middle school (n/a) | cafeteria | students, staff, and administra-tors | -- | -- | -- | -- | -- | peer-reviewed |
| **Wie S [17]** | 2003 | multiple methods - modeling, case studies, interviews | economics of labor, fees, and services | -- | observational (cross-sectional) | -- (--) | K-12 (110) | cafeteria | staff and administra-tors | 3,000 | -- | -- | -- | -- | peer-reviewed |

| | | | | | | | | | | | | | | | |
|---|---|---|---|---|---|---|---|---|---|---|---|---|---|---|---|
| **Smyth DP [40]** | 2010 | multiple methods - interviews, waste audit (sorted + weighed) | food packaging waste categorized: paper and paperboard, disposable hot beverage cups, beverage containers, plastics, expanded polystyrene, glass, ferrous metals, non-ferrous metals, organic material, hazardous by-products, electronic waste, non-recyclable other; level of MSW practices | trash, recycling, compost (food scraps) | observational (cross-sectional) | Canada (British Columbia) | college/ university (1) | whole school | students, staff, and administrators | 5,038 | -- | -- | -- | two 5-day waste audits | peer-reviewed |
| **Palmer S [32]** | 2021 | multiple methods - interviews, observations | food packaging waste categorized: packaging waste, recycling, food waste; level of MSW practices | trash | observational (cross-sectional) | US (CO) | K-12 (13) | cafeteria | staff and administrators | 20 | FRPM | -- | -- | -- | peer-reviewed |

| | | | | | | | | | | | | | | | |
|---|---|---|---|---|---|---|---|---|---|---|---|---|---|---|---|
| **Prescott MP [49]** | 2019 | multiple methods - food systems awareness poster analysis, question-naire before and after interventi on | level of food packaging knowledge | -- | experimental (pretest-posttest) | US (CO) | middle school (2) | cafeteria | students | 268 | ethnicity | formal educatio n | <1 month | -- | peer-reviewed |
| **Cunningha m-Scott CB [44]** | 2005 | multiple methods - curbside recycling reports, control compariso n and experimen t schools identified, interventi on, waste audits (sorted + weighed + got volume) before and after interventi on, question-naire, waste hauler reports | food packaging waste categorized: paper, food waste, carboard, comingled recyclables, non-recyclable trash; participation rates; level of food packaging knowledge | recyclin g (mixed paper), recyclin g (cans/bo ttles) | experimental (pretest-posttest) | US (TX) | elementar y school (3) | whole school | students, staff, and administra-tors | 558 | geograph ic informati on; ethnicity; English proficien cy | waste-reductio n or recycling adoption program; formal educatio n | 1 month - 1 year | 5-day waste audit | master's thesis |

| | | | | | | | | | | | | | | | |
|---|---|---|---|---|---|---|---|---|---|---|---|---|---|---|---|
| **Ward MN [18]** | 2014 | multiple methods - case study (tool formation), test case (informal interviews, intervention, waste audit (sorted + weighed) before and after intervention) | food packaging waste categorized: trash, paper, aluminum, plastics; level of MSW practices | trash, recycling (mixed paper), recycling (cans/bottles) | experimental (pretest-posttest) | US (NY) | elementary school (1) | whole school | students, staff, and administra-tors | -- | geographic information | assessment of recycling programs | <1 month | two 5-day waste audits | peer-reviewed |
| **Cagnassola L [7]** | 2016 | modeling | food packaging waste categorized: reusable trays, reusable utensils, plastic utensils, foam trays, compostable utensils, compostable trays | -- | observational (cross-sectional) | US (NY) | high school (1) | cafeteria | students | 450 | -- | -- | -- | -- | master's thesis |
| **Prestin A [19]** | 2010 | focus groups | level of knowledge, attitudes, and behaviors towards recycling | -- | observational (cross-sectional) | US (West Coast) | high school (--) | whole school | students | 62 | geographic information | -- | -- | -- | peer-reviewed |
